# Use of Weight-Management Mobile Phone Apps in Saudi Arabia: A Web-Based Survey

**DOI:** 10.2196/12692

**Published:** 2019-02-22

**Authors:** Ghadeer S Aljuraiban

**Affiliations:** 1 Department of Community Health Sciences College of Applied Medical Sciences King Saud University Riyadh Saudi Arabia

**Keywords:** lifestyle, mobile app, weight loss, mobile phone

## Abstract

**Background:**

In recent years, the use of mobile phone weight-management apps has increased significantly. Weight-management apps have been found effective in promoting health and managing weight. However, data on user perception and on barriers to app usage are scarce.

**Objective:**

This study aimed to investigate the use of weight-management apps and barriers to use as well as reasons for discontinuing use in a sample of mobile phone users in Saudi Arabia.

**Methods:**

Mobile phone users aged 18 years and above from the general public in Saudi Arabia completed a Web-based survey. The survey included questions on weight-management app usage patterns, user perceptions concerning weight management, efficacy of weight-management apps, and reasons for discontinuing use. Participants were classified into normal weight (body mass index [BMI]: 18.5 to 24.9 kg/m^2^) and overweight or obese (BMI: ≥25.0 kg/m^2^).

**Results:**

The survey included 1191 participants; 513 of them used weight-management apps. More overweight or obese respondents used these apps compared with normal weight respondents (319/513, 62.2% vs 194/513, 37.8%, respectively). App features that overweight or obese users were most interested in were mainly the possibility to be monitored by a specialist and barcode identification of calorie content, whereas normal weight users mostly preferred availability of nutrition information of food items. Reasons for discontinuing use among overweight or obese respondents were mainly that monitoring by a specialist was not offered (80/236, 33.9%) and the app was not in the local language (48/236, 20.3%). Among normal weight users, the main reason for noncontinuance was the app language (45/144, 31.3%) and difficulty of use (30/144, 20.8%).

**Conclusions:**

To better address the needs of both normal weight and overweight or obese adults, improved app designs that offer monitoring by a specialist are needed. Developers may consider ways of overcoming barriers to use, such as language, by developing local language apps, which can improve the efficacy of such apps and help spread their use.

## Introduction

### Background

Long-term analyses of trends in body mass index (BMI) levels show that obesity increased globally between 1980 and 2008 [1]. In 2013, a national survey reported that the prevalence of obesity in Saudi adults was 29% and was higher in women compared with men (33.5% vs 24.1%, respectively) [2]; by 2022, prevalence is expected to increase to 40% for men and 78% for women [3]. Several interventions including lifestyle and dietary modifications have been shown to be effective in the management of obesity; however, limitations such as financial cost and accessibility may restrict their use [4,5].

A recent, encouraging development is the marked increase in the number of mobile phone apps for weight loss or weight management [6-9]. According to the 2018 Global Mobile Market Report, Saudi Arabia has one of the highest mobile phone penetration rates in the world [10]. In 2017, Saudi Arabia had 21 million mobile phone users (84% of total adults); this is expected to increase to nearly 24 million in 2022 [11]. Mobile phone apps that focus on health have achieved worldwide popularity; over 325,000 health, fitness, and medical apps were available for download on all major app stores in 2017 [12].

With the projected epidemic in increasing obesity, frequent recording of both food intake and physical activity with weight-management apps may be a useful, potentially cost-effective and conveniently available method for modifying health-related behaviors [13]. A systematic review and meta-analysis of 12 randomized controlled trials and case-control studies showed that use of weight-management apps affected BMI by a net reduction of 0.4 kg/m^2^ more than control groups (ie, traditional interventions or intensive counseling) [14].

### Rational and Aim

Development and use of weight-management apps is still in an early phase, and sample populations in various countries have reported barriers such as limited functionality, lack of input options for tracking data, no evidence-based guidelines, and improper app classification (ie, only one-fifth of the apps classified as *health and fitness* or *medical* actually facilitated behavioral or physical activity; many simply offered health information) [15]. There is a paucity of evidence regarding usage and barriers of use in Saudi Arabia. One qualitative study restricted to Saudi women only reported that the available weight-management apps offered diet programs that lacked locally available foods and suggested physical activities that were not applicable to their environment [16].

Despite growing research on weight-management apps, evidence on user perspective—especially among users in Saudi Arabia, whose perspective varies vastly from that of the West—has been little explored. To the author’s knowledge, this is the first survey aimed to identify the sociodemographic characteristics of weight-management app users, app usage patterns, user perceptions, efficacy, and reasons for continuing or discontinuing use of a weight-management phone app in Saudi Arabia.

## Methods

### Design and Sample

From May to July 2018, mobile phone users aged 18 years and older from the general public in the city of Riyadh filled out an open Web-based survey. The survey was designed using *Microsoft* Forms (Microsoft Corp). Authentication cookies prevented multiple entries from the same individual. The survey adhered to advanced Web-based survey methodology, such as internet protocol address verification. The multifaceted recruitment strategy included university portals, social media, newsletters, and posters, all citing a quick response code for declaring interest. Those outside of the internet-based survey were not contacted. Of the 1302 visitors to the survey link, 1191 agreed to participate; all of the participants resided in Riyadh. Of these 1191, a total of 513 reported having previously downloaded an app to track their weight and completed all survey items. No personal identifying information was collected. The King Saud University Institutional Review Board approved all study procedures (reference # KSU-HE-18-1006).

### Survey Items

The survey comprised 28 items in the following domains: (1) sociodemographic characteristics (gender, age, employment, education, and income), (2) general health questions (weight, height, physical activity, smoking habits, and medical history), (3) usage patterns (number of apps used, frequency of use, reasons for use, and desired features), (4) user conceptions and efficacy of weight-management apps (effectiveness of weight-reduction apps, effectiveness of fitness apps, and effectiveness of long-term use), and (5) reasons for discontinuing use (number of apps that the respondent no longer uses and reasons for discontinuing use).

Krebs et al previously used the survey questions in their national survey throughout the United States [17]. As the survey items on app use had no known precedent, a team of experts with expertise in questionnaire design field-tested the items among researchers. The Web-based survey administration system was also pretested and then pilot-tested live for 1 day, attracting 86 respondents. These data, however, were not included in the final analysis of this study but were analyzed to adjust the Web-based survey for errors.

The items were presented in a logical order, and consideration was taken to reduce response set bias. Each question required a response before the respondent was allowed to advance to the next screen. The Web-based survey displayed 1 item per screen, with the first screen asking respondents for their informed consent to participate in the study. Following consent, participants were allowed to begin the survey. Participation was completely voluntary. A *back* button on each screen allowed participants to edit previous answers. The average time needed to complete the survey was about 8 min.

### Statistical Analysis

Statistical analyses were conducted using JMP (version 12; SAS Institute). No statistical correction procedures or weightings were applied. Categorical variables are presented as percentages, whereas continuous variables are presented as means and SDs. Participants were stratified by gender and by BMI, where participants were considered of normal weight if their BMI was 18.5 to 24.9 kg/m^2^ and overweight or obese if their BMI was ≥25 kg/m^2^.

Sample size was calculated based on the current population of Riyadh—the capital of Saudi Arabia has 5.5 million adults aged above 18 years [18] and 84% are mobile phone users [11]—using a confidence level of 95% and a precision of 5%. With these parameters, the minimum sample required for the analyses to have a power of 95% would be 207 individuals.

## Results

### Sociodemographic Characteristics and Health Status

Of the respondents, 43.07% (513/1191) reported that they had previously used an app to manage their weight; only analyses for weight-management app users are presented (Table 1). More overweight or obese respondents reported that they had previously used an app to track their weight compared with normal-weight respondents (319/513, 62.2% vs 194/513, 37.8%, respectively; Table 2). Most users were Saudi nationals (487/513, 94.9%), women (305/513, 59.5%), had a mean age of 26.6 (SD 8.1) years, and a mean BMI of 28.6 (SD 6.7) kg/m^2^. Most users were also educated and had a high monthly income. The majority of the female respondents were overweight or obese (178/305, 59.5%). Most users (399/513, 77.8%) considered their general health to be good, very good, or excellent. The analyses found gender differences in lifestyle: men were more likely to smoke than women, and more women reported that they never engaged in physical activity (Multimedia Appendix 1).

### Usage Patterns

Most weight-management app users used 1 to 5 apps (476/513, 92.8%; Table 2). The frequency of use was less than once a month for 34.9% (179/513) or a few times a week for 22.0% (113/513). In overweight or obese users, the most common reasons for wanting to download a weight-management app were to lose weight (134/319, 42.0%) and monitor food intake (88/319, 27.6%; Table 2). Normal-weight participants, on the other hand, downloaded a weight-management app mainly to monitor food intake (82/194, 42.3%) and track physical activity (52/194, 26.8%). The most common reasons for downloading a particular weight-management app were its rank in the app store, recommendations from friends and family (154/513, 30.0%), and social media influencers (93/513, 18.1%; Table 2).

The app features that overweight or obese users were most interested in were (1) the possibility to be monitored by a specialist (126/319, 39.5%), (2) barcode identification of calorie content (77/319, 24.1%), (3) availability of nutrition information on numerous food items (56/319, 17.6%), (4) a weekly or monthly progress report (44/319, 13.8%), and (5) constant reminders to follow a chosen diet or exercise (16/319, 5.0%). For normal-weight users, the desired features were (1) availability of nutrition information on numerous food items (67/194, 34.5%), (2) barcode identification of calorie content (54/194, 27.8%), (3) the possibility to be monitored by a specialist (31/194, 16.0%), (4) a weekly or monthly progress report (26/194, 13.4%), and (5) constant reminders to follow a chosen diet or exercise (16/194, 8.3%; Table 2). No gender differences in usage patterns were observed (Multimedia Appendix 2).

### User Conceptions and Efficacy of Weight-Management Apps

Most weight-management app users agreed or strongly agreed that apps were helpful in losing weight (Table 3). More normal-weight users agreed or strongly agreed that weight-management apps had helped them lose weight compared with overweight or obese users (98/194, 50.5% vs 148/319, 46.4%; Table 3). As for apps that suggested exercise plans, more normal-weight users agreed or strongly agreed that such apps helped them lose weight compared with overweight or obese respondents (116/194, 59.8% vs 177/319, 55.5%, respectively; Table 3).

Concerning efficacy, overweight or obese users were unsure whether weight-management apps would be effective in the long term (170/319, 53.3%), whereas normal-weight users agreed or strongly agreed that they were effective (102/194, 52.6%; Table 3). No gender differences were observed (Multimedia Appendix 3).

### Reasons for Discontinuing Use

Most users (380/513, 74.1%) reported that they have downloaded weight-management apps they no longer use, and the majority of these were overweight or obese (236/380, 62.1%; Table 4). Among overweight or obese users, reasons for discontinuing use included (1) monitoring by a specialist was not offered (80/236, 33.9%), (2) the app language was not the local language (48/236, 20.3%), (3) some costs of app usage were hidden (37/236, 15.7%), (4) the app was confusing to use (34/236, 14.4%), and (5) the respondent had lost interest (30/236, 12.7%). Normal-weight users stopped using an app because (1) it was not in the local language (45/144, 31.3%), (2) it was confusing to use (30/144, 20.8%), (3) it did not offer monitoring by a specialist, and (4) some costs were hidden (21/144, 14.6%). Reasons for discontinuing use were similar between men and women (Multimedia Appendix 4).

**Table 1:** Sociodemographic characteristics and health status of participants stratified by body mass index.

Characteristics		Body mass index		Total
		18.5 to 24.9 kg/m^2^	>25 kg/m^2^	
**Previously downloaded an *app* to track anything related to weight, n (%)**				
	No	283 (59.33)	395 (55.32)	678 (56.93)
	Yes	194 (40.67)	319 (44.68)	513 (43.07)
**Gender, n (%)**				
	Female	127 (65.46)	178 (55.80)	305 (59.45)
	Male	67 (34.54)	141(44.20)	208 (40.55)
**Nationality, n (%)**				
	Non-Saudi	8 (4.12)	18 (5.64)	26 (5.07)
	Saudi	186 (95.88)	301 (94.36)	487 (94.93)
Age (years), mean (SD)		24.4 (6.9)	27.7 (8.3)	—
**Employment, n (%)**				
	In school	122 (62.89)	161 (50.47)	283 (55.17)
	Not working or retired	7 (3.61)	20 (6.27)	27 (5.26)
	Working full-time	65 (33.51)	138 (43.26)	203 (39.57)
**Education, n (%)**				
	High school degree	42 (21.65)	45 (14.11)	87 (16.96)
	Bachelor’s degree	122 (62.89)	189 (59.25)	311 (60.62)
	Graduate degree (Masters, PhD, or MD)	30 (15.46)	85 (26.65)	115 (22.42)
**Household income, n (%)**				
	Less than 5,000 SR/month	7 (3.61)	20 (6.27)	27 (5.26)
	5,100 to 10,000 SR/month	55 (28.35)	93 (29.15)	148 (28.85)
	10,100 to 20,000 SR/month	76 (39.18)	105 (32.92)	181 (35.28)
	>20,000 SR/month	56 (28.87)	101 (31.66)	157 (30.60)
**General health status, n (%)**				
	Poor	2 (1.03)	14 (4.39)	16 (3.12)
	Average	32 (16.49)	66 (20.69)	98 (19.10)
	Good	22 (11.34)	43 (13.48)	65 (12.67)
	Very good	80 (41.24)	112 (35.11)	192 (37.43)
	Excellent	58 (29.90)	84 (26.33)	142 (27.68)
**Frequency of exercise or physical activity for at least 15 min in the past week, n (%)**				
	Never	55 (28.35)	119 (37.30)	174 (33.92)
	1 day	28 (14.43)	23 (7.21)	51 (9.94)
	2 days	39 (20.10)	49 (15.36)	88 (17.15)
	3-4 days	39 (20.10)	66 (20.69)	105 (20.47)
	5-7 days	33 (17.01)	62 (19.44)	95 (18.52)
**Overall nutritional status of the diet, n (%)**				
	Poor	22 (11.34)	80 (25.08)	102 (19.88)
	Fair	39 (20.10)	77 (24.14)	116 (22.61)
	Good	62 (31.96)	111 (34.80)	173 (33.72)
	Very good	59 (30.41)	45 (14.11)	104 (20.27)
	Excellent	12 (6.19)	6 (1.88)	18 (3.51)
**Smoking, n (%)**				
	Yes	23 (11.86)	53 (16.61)	76 (14.81)
	No	171 (88.14)	266 (83.39)	437 (85.19)
**Co-morbidities, n (%)**				
	None	135 (69.59)	212 (66.46)	347 (67.64)
	Hypercholesterolemia	9 (4.64)	8 (2.51)	17 (3.31)
	Hypertension	9 (4.64)	9 (2.82)	18 (3.51)
	Depression	8 (4.12)	46 (14.42)	54 (10.53)
	Diabetes	21 (10.82)	21 (6.58)	42 (8.19)
	Other chronic disease	12 (6.19)	23 (7.21)	35 (6.82)

**Table 2:** Pattern of use of weight-management apps stratified by body mass index.

Pattern of use		Body mass index		Total
		18.5-24.9 kg/m^2^	≥25 kg/m^2^	
**Number of weight-related phone apps used, n (%)**				
	1-5 apps	174 (89.6)	302 (94.6)	476 (92.79)
	6-10 apps	15 (7.73)	12 (3.76)	27 (5.26)
	>11 apps	5 (2.58)	5 (1.57)	10 (1.95)
**Frequency of weight-management app use, n (%)**				
	2 or more times a day	29 (14.95)	40 (12.54)	69 (13.45)
	About 1 time each day	17 (8.76)	32 (10.03)	49 (9.55)
	A few times each week	43 (22.16)	70 (21.94)	113 (22.03)
	A few times a month	43 (22.16)	60 (18.81)	103 (20.08)
	Less than once a month	62 (31.96)	117 (36.6)	179 (34.89)
**Reasons for downloading a weight-management app, n (%)**				
	Weight loss	26 (13.40)	134 (42.0)	160 (31.19)
	Monitor food intake	82 (42.27)	88 (27.59)	170 (33.14)
	Track how much activity or exercise I get	52 (26.80)	47 (14.73)	99 (19.30)
	Show or teach me exercises	19 (9.79)	24 (7.52)	43 (8.38)
	I want to kill time when bored	7 (3.61)	12 (3.76)	19 (3.70)
	Other reasons	8 (4.12)	14 (4.39)	22 (4.29)
**Reasons for downloading a particular weight-management app, n (%)**				
	Best ranked in the app store	53 (27.32)	101 (31.6)	154 (30.02)
	Recommendations from friends or family	66 (34.02)	88 (27.59)	154 (30.02)
	Social media influencers	32 (16.49)	61 (19.12)	93 (18.13)
	Web searches (eg, Google)	15 (7.73)	45 (14.11)	60 (11.70)
	Recommended by other apps	26 (13.40)	18 (5.64)	44 (8.58)
	TV	2 (1.03)	6 (1.88)	8 (1.56)
**Desired features of weight-management apps, n (%)**				
	Monitored by a specialist	31 (15.98)	126 (39.5)	157 (30.60)
	Can identify calories using barcode	54 (27.84)	77 (24.14)	131 (25.54)
	Nutrition information of many food items	67 (34.54)	56 (17.55)	123 (23.98)
	Provides a weekly or monthly progress report	26 (13.40)	44 (13.79)	70 (13.65)
	Reminders to follow a diet or exercise	16 (8.25)	16 (5.02)	32 (6.24)

**Table 3:** User conceptions and efficacy of weight-management apps stratified by body mass index.

User conceptions		Body mass index		Total
		18.5-24.9 kg/m^2^	>25 kg/m^2^	
**Apps that provide a weight-reduction meal plan helped in managing weight, n (%)**				
	Strongly disagree	65 (33.51)	74 (23.20)	139 (27.10)
	Disagree	11 (5.67)	19(5.96)	30 (5.85)
	Unsure	20 (10.31)	78 (24.45)	98 (19.10)
	Agree	82 (42.27)	125 (39.18)	207 (40.35)
	Strongly agree	16 (8.25)	23 (7.21)	39 (7.60)
**Apps that provide an exercise plan helped in managing weight, n (%)**				
	Strongly disagree	51 (26.29)	71 (22.26)	122 (23.78)
	Disagree	9 (4.64)	15 (4.70)	24 (4.68)
	Unsure	18 (9.28)	56 (17.55)	74 (14.42)
	Agree	96 (49.48)	148 (46.39)	244 (47.56)
	Strongly agree	20 (10.31)	29 (9.09)	49 (9.55)
**Weight-management apps are effective for long-term use, n** **(%)**				
	Strongly disagree	50 (25.77)	52 (16.30)	102 (19.88)
	Disagree	0 (0.00)	7 (2.19)	7 (1.36)
	Unsure	42 (21.65)	170 (53.29)	212 (41.33)
	Agree	80 (41.24)	60 (18.81)	140 (27.29)
	Strongly agree	22 (11.34)	30 (9.40)	52 (10.14)

**Table 4:** Reasons for discontinuing use, stratified by body mass index.

Reasons for discontinuing use		Body mass index		Total	
		18.5-24.9 kg/m^2^	>25 kg/m^2^		
**Have you downloaded any weight management apps that you no longer use? n (%)**					
	No	50 (25.77)	83 (26.02)		133 (25.93)
	Yes	144 (74.23)	236 (73.98)		380 (74.07)
**Why do you no longer use them? n (%)**					
	Monitoring by a specialist was not offered	21 (14.58)	80 (33.90)		101 (26.58)
	The app language was not in the local language	45 (31.25)	48 (20.34)		93 (24.47)
	There were hidden costs	21 (14.58)	37 (15.68)		58 (15.26)
	Loss of interest	17 (11.81)	30 (12.71)		47 (12.37)
	The app was confusing to use	30 (20.83)	34 (14.41)		64 (16.84)
	I no longer need it, or I met my goals	10 (6.94)	7 (2.97)		17 (4.47)

## Discussion

### Principal Findings

The survey found that only 43% of respondents used weight-management apps. Most users were young, educated women with a high income, and were overweight or obese. Overweight or obese users download a weight-management app mostly to lose weight, whereas normal-weight participants, on the contrary, download a weight-management app mainly to monitor food intake and track physical activity. The feature of the weight-management app that overweight or obese users desired most was the possibility to be monitored by a specialist, whereas the least desired feature was the constant reminders to follow a diet or exercise. On the contrary, normal-weight participants mainly desired apps that could provide nutrition information and calorie content of food items through barcode identification.

Among all users of a weight-management app, this study found that one of the main reasons for discontinuing use was that the language of the app was not the local language of the user. Among overweight or obese users, another main reason was lack of monitoring by a specialist, and among normal-weight users, difficulty of use.

### Comparison With Previous Work

In this study, 43% of the study sample currently used weight-management apps. A similar survey by Krebs and Duncan showed health app use among US adults to be about 60% [17]. Cultural differences and the newness of app use for health and weight management in the Saudi population may explain the difference [19].

Findings regarding the characteristics of weight-management app users in our survey are consistent with the results of a previous large-scale study in the United States [20]; we also found that most participants were young, had a higher income, had higher education, and reported very good or excellent health. Age and education were found to be important predictors for using mobile phones and apps as younger people are more exposed to mobile technology than the elderly, resulting in a higher utilization of mobile health-related apps [21]. We also found that the highest use of weight-management apps was among women. Women’s high use of weight-management apps in our study is in line with previous investigations that showed women were more concerned about their health status and tended to follow a healthier dietary pattern than men [22]. We also found more overweight or obese participants used such apps (mostly to lose weight) compared with normal-weight participants, who mostly wanted to maintain their weight. This is consistent with a previous study showing that weight-management apps were mostly downloaded by overweight or obese users, which may be because these users are more concerned about their overall health than others [23].

We found that overweight or obese participants mostly preferred to be monitored by a specialist compared with other features of the app such as constant reminders to follow a diet or exercise. A previous intervention found that, for encouraging healthy behavior, individually tailored feedback and action plans were preferable to general health instructions [24]. A randomized controlled trial showed that individualized health apps were more effective in addressing patients’ needs and provided the high-quality information and support required by patients [25]. In our survey, the most frequent reason for downloading a weight-management app, as reported by overweight or obese users, was to reduce caloric intake, whereas normal-weight users wanted to monitor their food intake. Among US health-related app users, Krebs et al found that the main reason for downloading apps was to track how much physical activity they were getting (52.8%) [17]. Their findings, however, did not differentiate between normal weight and overweight or obese users. The difference in preference may be related to cultural and social variables in Saudi Arabia rather than biological reasons, although activities such as walking for women is generally acceptable in major cities; this is not always the case in rural parts of the country [26].

As for efficacy of use, more normal-weight users found that weight-management apps helped them lose weight compared with overweight or obese users. A randomized controlled trial, however, found that apps for weight loss did not affect the weight of overweight primary care patients and were only useful for individuals who were willing to self-monitor their calories [27].

Participants in our survey also tended to discontinue use of the app for various reasons such as monitoring by a specialist was not offered and app was not in the local language. Similar barriers were reported in a previous qualitative study on the challenges of app use in supporting health behavior changes, showing that users generally found health apps to be time consuming, have unexpected costs, and lack the possibility of consulting an expert [28]. A previous qualitative study restricted to Saudi women only reported that only a few obese or overweight women were aware of the availability of weight-management apps and reported barriers of app use to be lack of motivational support and the language of the app [16].

### Strengths and Limitations

The strength of this study is that it is the first of its kind to target app users in Saudi Arabia. Additionally, it provides information on the demographics of this particular region along with insight into the extent of weight-management app use and barriers of use, which varies from barriers in other regions. This study also highlighted that weight-management apps are not only used by overweight or obese adults but also by normal-weight adults who wish to maintain their weight. Data on the differences in perception of use between overweight and normal-weight adults are also provided.

Although this is the first study to characterize user perspective on weight-management apps in Riyadh, Saudi Arabia, there were a few limitations. The main limitation was study design; a causal relationship cannot be inferred, and usage patterns may change over time. Second, the study sample included the city of Riyadh only and was not representative of the general population. A third limitation was the self-recorded weight and height of participants, which is a possible source of error. Finally, as this was a convenience sample, it did not include individuals who are overweight or obese but do not own mobile phones, although a large percentage of the population use mobile phones.

### Conclusions

Although the majority of users believe that weight-management apps are efficacious, considerable challenges remain. Given the increasing prevalence of overweight and obesity in Saudi Arabia and the high accessibility to mobile phones among adults, app developers should address findings of this study, especially regarding reasons for use and discontinuance. More specifically, app developers should consider the aforementioned barriers by developing apps in local languages and by developing apps which are linked to health professionals to provide individualized plans to manage the weight of both normal and overweight adults.

## Appendix

Multimedia Appendix 1 Sociodemographic characteristics and health status of participants stratified by gender.

Multimedia Appendix 2 Pattern of use of weight-management apps stratified by gender.

Multimedia Appendix 3 User conceptions and efficacy of weight-management apps stratified by gender.

Multimedia Appendix 4 Reasons for discontinuing use stratified by gender.

## Abbreviations

BMI: body mass index
