# Supplementary material for: Use of Weight-Management Mobile Phone Apps in Saudi Arabia: A Web-Based Survey
Source: JMIR Mhealth Uhealth. 2019 Feb 22;7(2):e12692. doi: 10.2196/12692 (PMC6406230; doi:10.2196/12692)
Supplement: Multimedia Appendix 1 [file mhealth_v7i2e12692_app1.pdf]

Multimedia Appendix 1. Sociodemographic Characteristics and Health Status of Participants Stratified by Gender[a]

	Females		Males	
	N	%	N	%
**Previously downloaded an "app" to track anything related to weight**				
No	376	55.21	302	59.22
Yes	305	44.79	208	40.78
**Participants who responded "yes" to app use**				
**Nationality**				
Non-Saudi	14	4.59	12	5.77
Saudi	291	95.41	196	94.23
**Employment**				
Student	160	52.46	123	59.13
Not working or retired	22	7.21	5	2.40
Working full-time	123	40.33	80	38.46
**Education**				
High school degree	50	16.39	37	17.79
Bachelor's degree	187	61.31	124	59.62
Graduate degree (Masters, PhD, MD, etc.)	68	22.30	47	22.60
**Household income**				
Less than 5,000 SR/month	17	5.57	10	4.81
5,100 to 10,000 SR/month	103	33.77	45	21.63
10,100 to 20,000 SR/month	101	33.11	80	38.46
>20,000 SR/month	84	27.54	73	35.10
**In general, would you say your health is:**				
Poor	7	2.30	9	4.33
Average	56	18.36	42	20.19
Good	43	14.10	22	10.58
Very good	127	41.64	65	31.25
Excellent	72	23.61	70	33.65
**Frequency of exercise or physical activity for at least 15 minutes in the past week**				
Never	100	32.79	74	35.58
1 day	36	11.80	15	7.21
2 days	61	20.00	27	12.98
3-4 days	75	24.59	30	14.42
5-7 days	33	10.82	62	29.81
**Overall health status of the diet**				
Poor	51	16.72	51	24.52
Fair	85	27.87	31	14.90
Good	110	36.07	63	30.29
Very good	52	17.05	52	25.00
Excellent	7	2.30	11	5.29
**Do you smoke cigarettes?**				
Yes	22	7.21	54	25.96
No	283	92.79	154	74.04
**Co-morbidities**				

None	211	69.18	136	65.38
Hypercholesterolemia	7	2.30	10	4.81
Hypertension	12	3.93	6	2.88
Depression	36	11.80	18	8.65
Diabetes	26	8.52	16	7.69
Other chronic disease	13	4.26	22	10.58

[a] All data are percentages unless otherwise noted
