# Supplementary material for: Use of Weight-Management Mobile Phone Apps in Saudi Arabia: A Web-Based Survey
Source: JMIR Mhealth Uhealth. 2019 Feb 22;7(2):e12692. doi: 10.2196/12692 (PMC6406230; doi:10.2196/12692)
Supplement: Multimedia Appendix 2 [file mhealth_v7i2e12692_app2.pdf]

Multimedia Appendix 2. Pattern of Use of Weight-Management Apps Stratified by Gender[a]

	Females		Males	
	N	%	N	%
**Number of weight-related phone apps used**				
1-5 apps	277	90.82	199	95.67
6-10 apps	21	6.89	6	2.88
>11 apps	7	2.30	3	1.44
**Frequency of using the weight management app**				
2 or more times a day	39	12.79	30	14.42
About 1 time each day	33	10.82	16	7.69
A few times each week	60	19.67	53	25.48
A few times a month	70	22.95	33	15.87
Less than once a month	103	33.77	76	36.54
**Reasons for wanting to download a weight management app**				
Weight loss	114	37.38	46	22.12
Monitor food intake	100	32.79	70	33.65
Track how much activity/exercise I get	39	12.79	60	28.85
Show/teach me exercises	29	9.51	14	6.73
I want to kill time when bored	10	3.28	9	4.33
Other Reasons	13	4.26	9	4.33
**Reason for downloading a particular weight-management app**				
Best ranked in the app store	77	25.25	77	37.02
Recommendations from friends or family	82	26.89	72	34.62
Social Media influencers	73	23.93	20	9.62
Web searches (e.g. Google)	40	13.11	20	9.62
From other apps	28	9.18	16	7.69
TV	5	1.64	3	1.44
**Desired features of weight management apps**				
Monitored by a specialist	94	30.82	63	30.29
Can identify calories using barcode	77	25.25	54	25.96
Nutrition Information of many food items	90	29.51	33	15.87
Provides a Weekly or monthly progress report	28	9.18	42	20.19
Constant reminders to follow the diet or exercise	16	5.25	16	7.69

[a] All data are percentages unless otherwise noted
