# Supplementary material for: Use of Weight-Management Mobile Phone Apps in Saudi Arabia: A Web-Based Survey
Source: JMIR Mhealth Uhealth. 2019 Feb 22;7(2):e12692. doi: 10.2196/12692 (PMC6406230; doi:10.2196/12692)
Supplement: Multimedia Appendix 3 [file mhealth_v7i2e12692_app3.pdf]

Multimedia Appendix 3. User Conceptions and Efficacy of Weight-Management Apps Stratified by Gender[a]

	Females		Males	
	**N**	**%**	**N**	**%**
**Apps that provide a weight reduction meal plan helped in managing weight**				
Strongly disagree	75	24.59	64	30.77
Disagree	24	7.87	6	2.88
Unsure	40	13.11	58	27.88
Agree	134	43.93	73	35.10
Strongly agree	32	10.49	7	3.37
**Apps that provide an exercise plan helped in managing weight**				
Strongly disagree	53	17.38	69	33.17
Disagree	17	5.57	7	3.37
Unsure	36	11.80	38	18.27
Agree	164	53.77	80	38.46
Strongly agree	35	11.48	14	6.73
**Weight management apps are effective for long term use**				
Strongly disagree	50	16.39	52	25.00
Disagree	2	0.66	5	2.40
Unsure	100	32.79	112	53.85
Agree	111	36.39	29	13.94
Strongly agree	42	13.77	10	4.81

[a] All data are percentages unless otherwise noted
