# Supplementary material for: Use of Weight-Management Mobile Phone Apps in Saudi Arabia: A Web-Based Survey
Source: JMIR Mhealth Uhealth. 2019 Feb 22;7(2):e12692. doi: 10.2196/12692 (PMC6406230; doi:10.2196/12692)
Supplement: Multimedia Appendix 4 [file mhealth_v7i2e12692_app4.pdf]

Multimedia Appendix 4. Reasons for Discontinuing Use Stratified by Gender[a]

	Females		Males	
	**N**	**%**	**N**	**%**
**Are there any health apps you downloaded and no longer use?**				
No	88	28.21	45	22.39
Yes	224	71.79	156	77.61
**What reasons do you no longer use them**				
Monitoring by a specialist was not offered	61	27.23	40	25.64
The app language is not in the local language	63	28.13	30	19.23
Were hidden costs	38	16.96	20	12.82
Loss of interest	24	10.71	23	14.74
Confusing to use	29	12.95	35	22.44
No longer need it/ I met my goals	9	4.02	8	5.13

[a] All data are percentages unless otherwise noted
